# Effect of propofol on heart rate and its coupling to cortical slow waves in humans

**DOI:** 10.1097/ALN.0000000000004795

**Published:** 2024-01-01

**Authors:** Marco S. Fabus, Jamie W. Sleigh, Catherine E. Warnaby

**Affiliations:** 1 Wellcome Centre for Integrative Neuroimaging, FMRIB Centre, Nuffield Department of Clinical Neurosciences, University of Oxford, Oxford, United Kingdom; 2 Nuffield Division of Anaesthetics, University of Oxford, Oxford, United Kingdom; 3 Department of Anaesthesiology, Faculty of Medical and Health Sciences, University of Auckland, Auckland, New Zealand

**Keywords:** propofol, electrocardiogram, heart rate, slow waves, electroencephalography, heart-brain interactions, cortico-cardiac coupling

## Abstract

**Background:**

Propofol causes significant cardiovascular depression and a slowing of neurophysiological activity. However, literature on its effect on the heart rate remains mixed, and it is not known whether cortical slow waves are related to cardiac activity in propofol anesthesia.

**Methods:**

We performed a secondary analysis of ECG and EEG data collected as part of a previously published study where N=16 healthy volunteers underwent a slow infusion of propofol up to an estimated effect-site concentration of 4 μg/ml. Heart rate, heart rate variability, and individual slow EEG waves were extracted for each subject. Timing between slow-wave start and the preceding R-wave was tested against a uniform random surrogate. Heart rate data were further examined as a post hoc analysis in N=96 ASA-2/3 older clinical population collected as part of the AlphaMax trial.

**Results:**

The slow propofol infusion increased the heart rate in a dose-dependent manner (increase of +4.2±1.5 bpm/(μg ml^-1^), P<0.001). The effect was smaller but still significant in the older clinical population. In healthy volunteers, propofol decreased the ECG R-wave amplitude (decrease of -83 [-245, -28] μV, P<0.001). Heart rate variability showed a loss of high-frequency parasympathetic activity. Individual cortical slow waves were coupled to the heartbeat. Heartbeat incidence peaked about 450ms before slow-wave onset and mean slow-wave frequency correlated with mean heart rate.

**Conclusions:**

We observed a robust increase in heart rate with increasing propofol concentrations in healthy volunteers and patients. This was likely due to decreased parasympathetic cardio-inhibition. Similar to non-rapid eye movement sleep, cortical slow waves are coupled to the cardiac rhythm, perhaps due to a common brainstem generator.

## Introduction

Propofol is the most widely used intravenous anesthetic hypnotic drug due to its favorable kinetics, low adverse effects incidence, and smooth induction profile^1^. It also causes significant cardiovascular depression, manifesting mainly as arterial hypotension^2^. However, despite decades of use, the effect of propofol on heart rate (HR) remains controversial. In clinical settings, propofol administration has been reported to carry a risk of bradycardia^1,3^ and several texts state that propofol decreases the heart rate as an accepted fact^4,5^. Others however find propofol to have no effect on the heart rate^2,6^, and much of the literature, especially in laboratory settings, appears to show significant *increases* in heart rate^7–12^. Clinical research is complicated by common co-administration of opioids with some bradycardic effects. On theoretical grounds, propofol’s heart rate effect may be due to modulation of GABAergic neurotransmission to cardiac parasympathetic neurons in the brainstem^11^.

Propofol also affects autonomic nervous activity. Because the autonomic nervous system regulates the heart rate, this can be indexed by heart rate variability, the beat-to-beat variation in heart rate (distinct from the mean heart rate, HR). ‘High frequency’ (~0.5 s to 7s) fluctuations in heart rate variability are dominated by parasympathetically mediated respiratory sinus arrythmia and are a measure of vagal tone. Propofol decreases short-term heart rate variability, in part through this lowered parasympathetic tone^6^. Lower-frequency fluctuations (seconds to tens of seconds) are driven by other, largely sympathetic, factors.

In the brain, propofol causes neuronal hyperpolarization by prolonging GABA-activated chloride channel opening. At the network level, this causes the cortex to switch between high-activity up states and silent down states. This switching can be observed as slow (~1Hz) waves on the electroencephalogram^13^. As propofol dose increases, power in the slow-wave band (typically 0.5-1.5Hz) saturates and the thalamocortical system becomes isolated from environmental stimuli^8^. By disrupting cortical information processing, slow waves may have a causal role in sustaining unconsciousness^14^. Similar slow waves are observed in non-rapid eye movement (NREM) sleep^15^ where they have been linked to changes in autonomic activity including individual heartbeats^16–18^.

In this study, we first performed an advanced secondary analysis of electroencephalographic (EEG) and electrocardiographic (ECG) data collected in N=16 healthy volunteers undergoing a slow propofol infusion up to 4μg/ml estimated effect-site concentration. As effects of propofol may depend on induction speed^19^, our ultra-slow infusion provides a unique perspective without influences of concomitant medication. We hypothesized that propofol would increase heart rate and decrease parasympathetic effects, as indexed by high-frequency heart-rate variability. Our secondary aim was to explore a possible link between ECG activity and the frontal cortical slow waves seen in the EEG. We hypothesized that, like NREM sleep, slow waves would preferentially occur time-locked to individual heart beats. Finally, to see if our heart rate findings held clinical validity, we also performed a post-hoc analysis of clinical EEG and HR data from N=96 ASA-2/3 AlphaMax study patients^20^.

## Materials and Methods

### Data collection

32-channel EEG and single-channel ECG was collected in N=16 healthy subjects (8 female, age 28.6±7 years; Table 1) during slowly increasing intravenous infusion of propofol up to an estimated effect-site concentration of 4 μg/ml using the Marsh pharmacokinetic model^8^. The experiment was separated into 4 main periods: 10 minutes awake, 48 minutes induction, 10 minutes peak anesthesia and 48 minutes emergence. Informed written consent was obtained from all participants; details of this experiment have been published previously^8^.

### Data pre-processing

EEG data pre-processing was carried out with BrainVision Analyzer version 2.1 (BrainProducts GmbH), custom written MATLAB code (Matlab 2019b, Math Works Inc.), and the EEGLAB (v2019.1) analysis toolbox. The EEG and ECG data were re-referenced to the common average of signals from all EEG channels. This was done as theoretical reasons suggest scalp average to be a robust null reference which decreases volume conduction effects^21^. Independent component analysis and bad channel rejection was performed to remove EEG data with blinks and ocular movements. EEG data were band-pass filtered with a phase-preserving third order 0.5Hz-45Hz Butterworth filter. EEG data was down-sampled to 100Hz and ECG to 500Hz.

### Time-series ECG analysis

Heart rate, ECG waveform templates, and R-wave amplitudes were extracted using the BioSPPy toolbox (https://github.com/PIA-Group/BioSPPy/) which uses Hamilton segmentation^22^. This was used on each subject to identify individual R-wave peaks, the heart rate, and ECG waveform templates, which were subsequently Spearman-correlated to the propofol effect-site concentration.

### Heart rate variability ECG analysis

In order to explore correlates of autonomic activity, standard heart rate variability metrics were extracted for 5-minute segments in each subject using the pyHRV toolbox^23^ and Spearman-correlated with propofol effect-site concentration at the group level. These heart rate variability metrics included root-mean-square successive difference between R peaks as well as other frequency domain metrics. Specifically, the frequency domain metrics used were the ratio of low-frequency (LF; largely sympathetic, 0.04Hz-0.15Hz) and high-frequency (HF; parasympathetic, 0.15Hz-0.4Hz) heart rate variability and the peak frequency in the high frequency band. The low to high frequency ratio is thought to index the balance between sympathetic and parasympathetic activity, with a low LF/HF reflecting parasympathetic dominance, though this simple interpretation has been challenged^24^.

### Slow-wave analysis

Slow-wave activity was defined as the Fourier power in the 0.5Hz-1.5Hz band on the frontal Fz channel, as is common convention^8^. The correlation between slow-wave activity and heart rate / effect-site concentration and its significance was found using Spearman correlation in 5-minute segments. Individual slow waves were then identified using standard methodology based on amplitude and duration thresholding implemented in the yasa toolbox^15,16,25^. In brief, each slow wave had to have amplitude in the 99^th^ percentile of the 0.5-4Hz amplitude and negative duration between 0.25s and 1.25s. This wider filter is commonly chosen to capture more details of the non-sinusoidal slow wave shape^26^, though proposals for multiple slow wave types exist^27^. Slow wave onset was defined as the initial downward zero crossing and slow-wave frequency was extracted as the inverse of the slow-wave period.

### Cortico-cardiac coupling analysis

Once heartbeats and slow waves were identified, we aimed to test whether heartbeats occurred at preferential times in the slow wave cycle. For each slow wave detected, the time delay relative to the slow wave start (initial downward zero crossing) was noted for 8 heartbeats closest to it. Eight beats were chosen as this window length fully covers aslow wave. This resulted in 8 R-wave to slow wave (RS) intervals, using methodology similar to previous cardio-respiratory analyses^28,29^. We wanted to know if ECG R-wave to EEG slow wave timings were distributed randomly or in phase with the slow wave onset. For robustness, this was tested against a surrogate null distribution in several ways. First, we utilized the same method that has previously been used to study cardiorespiratory coupling^30^. This method compares the RS_-1_ interval (i.e. the time interval between slow-wave start and the preceding R-wave peak) to a uniformly random null distribution. Starting from the beginning of each subject’s set of RS_-1_ values, we used a moving window of 40 slow waves, and placed the corresponding RS-1 intervals in a 10-bin histogram with outer limits of 0 and the mean heart period for that window. From the histogram, the proportional Shannon entropy is calculated as follows:

$$\begin{array}{c} \text{Shannon}\;\text{entropy}=\text{SH}=\sum_{b=1}^{N}P_{b}\times \log P_{b}\\ \text{Maximum}\;\text{Shannon}\;\text{entropy}={\text{SH}}_{\max}=-\log\frac{1}{N}\\ \text{Proportional}\;\text{Shannon}\;\text{entropy}={\text{SH}}_{\text{P}}=\text{SH}/{\text{SH}}_{\max}\text{,} \end{array}$$

where P_b_ is the histogram probability of bin *b* and *N* is the number of histogram bins.

During perfect coupling, all RS_-1_ intervals fall into one bin and SH_p_=0. In the absence of coupling, RS-1 intervals are distributed randomly, producing maximum entropy with SH_p_=1. For each subject, the mean SH_p_ across the whole experiment was computed. To determine a significance threshold, SH_P_ was computed for N=10,000 surrogate series of 200 random numbers each, drawn from a uniform distribution between 0 and 1 (mean heart rate of 60bpm). The 0.1^st^ percentile was used to indicate significance at the P=0.001 level (SH_P_=0.970). Additional methods testing autocorrelation of the RS histogram and more complex surrogates were used to further verify robustness of this result (see Supplemental Digital Content).

Additionally, for each slow wave identified, ±2s of EEG and ECG activity were saved around the slow wave start. This was then averaged across slow waves and subjects to reveal any coherent ECG patterns during a slow wave.

### Clinical dataset analysis

In order to carry out a preliminary exploration of whether our heart rate results could be replicated in clinical data, we performed a post-hoc analysis of heart rate and drug concentrations in N=96 patients collected as part of the AlphaMax study (median age 74yrs (range 61 to 86yrs), 66 male, ASA 2/3, variety of procedures; Table 1). This dataset contained EEG, drug concentration, heart rate, and demographic data. Unfortunately, individual ECG waveforms were not available in this dataset, so we could not determine the heart rate variability or cortico-cardiac coupling analyses.

The AlphaMax study patients received a standardized desflurane and fentanyl-based maintenance general anesthesia that was titrated to maximize the EEG alpha power in the intervention group. For each patient, heart rate, and drug concentrations (propofol, fentanyl, desflurane) were sampled – or estimated using population based pharmacokinetic models – every 5 seconds. The heart rate was smoothed with a 2min moving median window to suppress artifacts and any heart rate above 250bpm or below 10bpm was not used. A large mixed-effects general linear model was constructed with heart rate, drug concentrations, and demographic variables as regressors. Specifically, the fixed effects of propofol, fentanyl, and desflurane (+ their linear interaction terms), as well as age, BMI, ASA status (2 or 3), and sex were studied. A random effect of each individual’s mean heart rate was included. In summary, the model equation was

$$\text{HR}[\text{bpm}]=\beta_{0}+\beta_{1}{}^{*}\text{age}+\beta_{2}{}^{*}\text{BMI}+\beta_{3}{}^{*}\text{ASA}+\beta_{4}{}^{*}(\text{sex}=\text{F})+\beta_{5}{}^{*}\text{prop}+\beta_{6}{}^{*}\text{fent}+\beta_{7}{}^{*}\text{des}+\beta_{8-}{}^{*}{\text{prop}}^{*}\text{fent}+\beta_{9}{}^{*}{\text{prop}}^{*}\text{des}+\beta_{10}{}^{*}{\text{fent}}^{*}\text{des}+(1|\text{patient}\;\text{number}),$$

where β_i_ are the model coefficients, and prop=propofol [μg/ml], des=desflurane [%], and fent=fentanyl [ng/ml] effect-site concentrations. To compare possible cortico-cardiac interactions with the propofol dataset, individual slow waves were extracted from intraoperative EEG (from first incision to the end of closing up) and mean slow-wave frequency per subject extracted and compared with the mean heart rate.

### Statistical analyses

As these analyses were all post hoc analyses of previously collected and published data, no power calculation was done. Spearman correlations and their P-values were used to test associations between ECG/EEG parameters (heart rate, R-wave amplitude, root-mean-square successive difference, low frequency to high frequency ratio, peak high frequency, slow-wave power) and propofol concentration. Repeated-measures analysis of variance (RM-ANOVA) was performed on ECG/EEG-derived parameter traces in 5-minute segments to further test for significant changes. For display purposes, mean ± standard error across participants is shown, except where the data was not normally distributed (as tested with D’Agostino and Pearson’s test). In these non-normally distributed cases, median ± bootstrapped 95% confidence interval (10,000 iterations) are shown. Mean ± SD (or median [25^th^, 75^th^ percentile] are given in the text. Significance was set at the P=0.05 level unless otherwise specified. All custom code used in this study is available at https://gitlab.com/marcoFabus/fabus2022brainheart.

## Results

### Time-series ECG analysis

**Figure 1:**
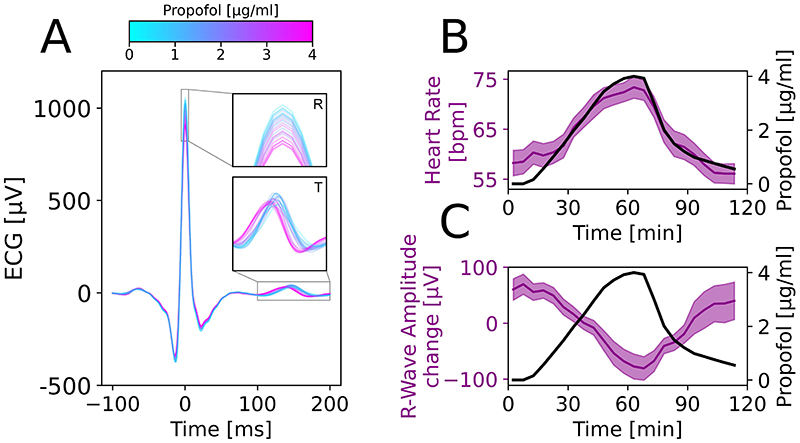
Propofol increased heart rate and changed ECG shape in healthy volunteers. (A) Mean ECG waveform for all heart beats across N=16 subjects across propofol doses. High propofol concentration (pink) was characterized by a decrease in R-wave amplitude (top inset) and an earlier T-wave (lower inset). (B) Propofol increased the heart rate. Group-level heart rate results (purple; mean ± SEM) and propofol effect-site concentration (black; Spearman ρ=0.923, P<0.001). (C) At the group level, R-wave amplitude (RWA; purple; mean ± SEM) inversely tracked propofol concentration (black; Spearman ρ=−0.902, P<0.001).

First, we tracked the heart rate and time-series ECG properties across an ultraslow propofol induction and emergence in N=16 healthy volunteers (Figure 1). At higher propofol doses, we observed a shortening of the QT segment and decrease in R-wave amplitude (Figure 1A). In every subject, the heart rate increased and very robustly tracked the propofol dose with Spearman correlation of ρ=0.923, P<0.001 (Figure 1B). Mean heart rate across volunteers increased from 58.2±10bpm at baseline to 73.4±8.8bpm at peak anesthesia, equivalent to an increase of 4.2±1.5 bpm/(μg ⋅ml^-1^). The maximum effect size comparing heart rate at baseline and peak propofol was Cohen’s d=1.546. A linear regression showed the heart rate and propofol relationship to be HR [bpm] = 56.1 (54.9, 57.2) + 4.23 (3.75, 4.80) * propofol [μg/ml], where brackets show 95% confidence intervals. Similarly, the R-wave amplitude was also strongly inversely correlated with the propofol effect-site concentration (Spearman ρ=−0.902, P<0.001, Figure 1C). R-wave amplitude decreased from 966 [707, 1133] μV at baseline to 742 [627, 1068] μV at peak anesthesia, equivalent to a decrease of -83 [-245, -28] μV.

### Heart rate variability analysis

**Figure 2:**
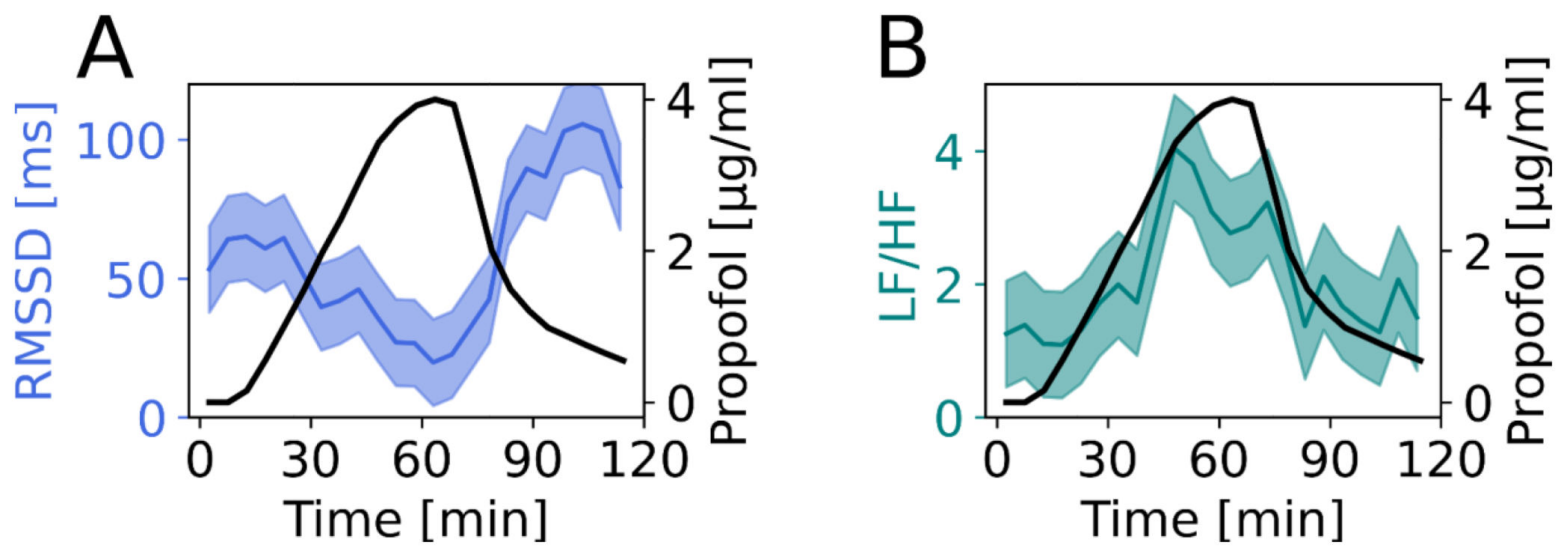
Propofol decreased parasympathetic activity, as indexed by heart rate variability in healthy volunteers. (A) Root-mean-square successive difference between heart beats, a measure of parasympathetic activity, (blue; median ± 95% CI) inversely tracks propofol concentration and rebounds on emergence (black). (B) Low frequency to high frequency ratio (green; median ± 95% CI), a metric thought to index balance of sympathetic and parasympathetic activity shows an increase with propofol (black).

Next, we studied autonomic activity through heart-rate variability (HRV; Figure 2). The root-mean-square successive difference (RMSSD) between heartbeats, which indexes parasympathetic tone, decreased in proportion to propofol concentration, and rebounded on emergence (Figure 2A; Spearman ρ=−0.785, P<0.001, Cohen’s d=1.296 for baseline vs peak concentration). This was confirmed by a repeated measures ANOVA (RM-ANOVA) across subjects with significance P<0.001.

With regard to the frequency domain metrics, the low to high frequency ratio showed higher between-subject variability, but the group average confirmed the shift towards a relative predominance of sympathetic activity with increasing propofol concentration (Figure 2B; Spearman ρ=−0.763, P<0.001). The associated RM-ANOVA result also showed a significant change (P=0.003, Cohen’s d=0.539) between baseline and highest propofol concentration. The peak frequency in the high-frequency parasympathetic HRV range also tracked with propofol concentrations (Supplementary Figure 7; Spearman ρ=0.885, P<0.001; RM-ANOVA P<0.001).

### Slow-wave analysis

**Figure 3:**
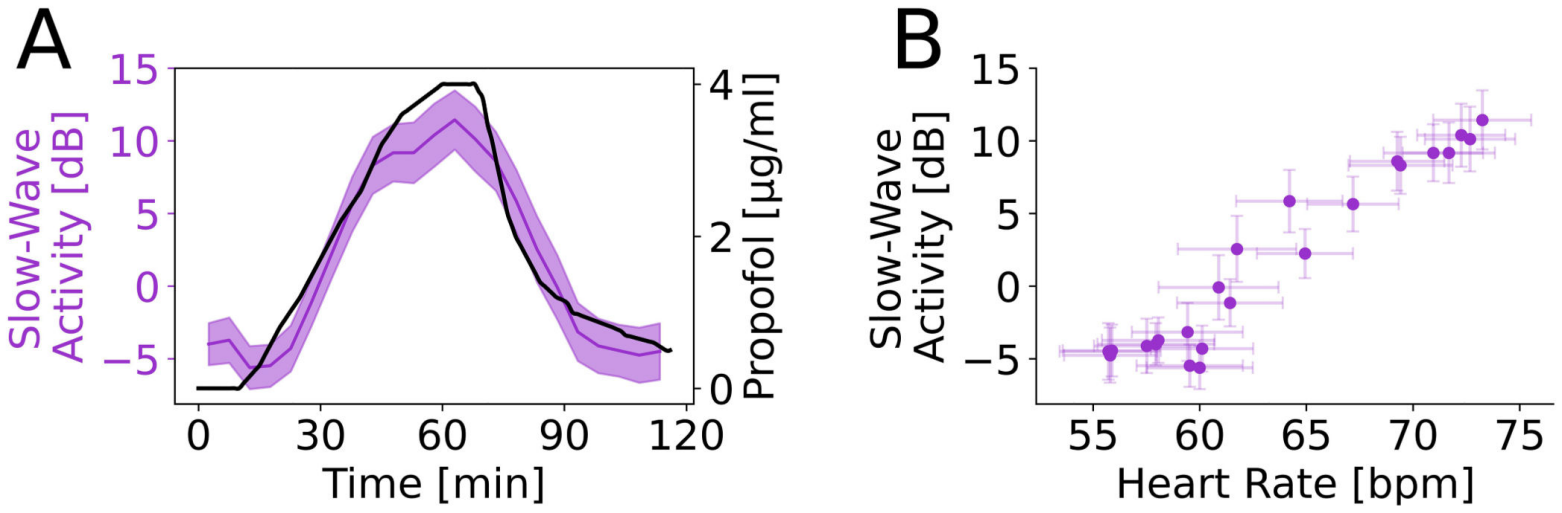
Propofol increased frontal cortical slow-wave activity and this increase tracked heart rate increases in healthy volunteers. (A) Group-level Fz slow-wave activity results (purple; mean ± standard error) against propofol concentration (black). Slow-wave activity increases and plateaus with drug dose. (B) On the group level, increases in heart rate saw corresponding increases in slow-wave power. Each purple dot represent a 5-minute segment of the experiment with standard errors across subjects shown.

Cortical activity during propofol anesthesia is known to be associated with non-REM deep sleep-like slow-wave activity (Figure 3). We first confirmed the previous finding of saturation of frontal slow-wave activity with propofol dose at the Fz electrode^8^ (Figure 3A). However, more strikingly, this slow-wave activity increase correlated very strongly with the increasing heart rate (Figure 3B; Spearman ρ=0.910, P<0.001).

### Cortico-cardiac coupling

**Figure 4:**
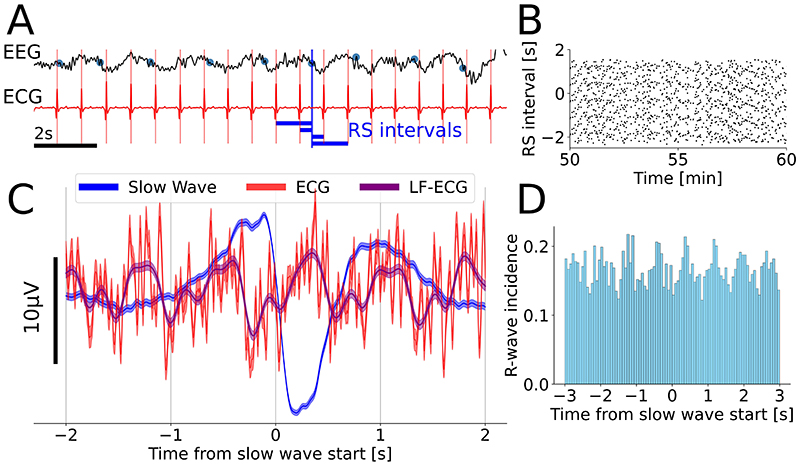
Single-subject low-frequency cortico-cardiac coupling. (A) Example 15s of EEG and ECG data at high propofol concentrations. Slow-wave starts are shown with blue dots. For each slow-wave start, duration of intervals to nearest heartbeats is determined (R-S intervals, R-wave/Slow wave). Individual R-waves are marked with red vertical lines. (B) Example illustrative raster plot of RS intervals during 10 minutes of peak anesthesia. Heartbeats are not distributed randomly and show modest horizontal clustering in horizontal lines, demonstrating non-random coupling to slow-wave onset (proportional entropy SHP=0.890). (C) Single-subject average slow wave (blue; mean ± SEM across all slow waves) and ECG amplitudes (red = broadband, purple = 0.5Hz-1.5Hz only) time-locked to slow-wave onset. The ECG pattern is not uniform random noise but shows a clear ongoing low-frequency ECG oscillation. (D) Histogram of R-wave timings relative to slow-wave onset. Individual heartbeats preferentially occur in phase with the slow wave, explaining the oscillatory appearance of (C) and stripes seen in (B).

The identified correlation between slow-wave activity and heart rate, as well as previous literature describing their coupling in sleep, led us to focus on quantifying the presence of any time-related coupling between individual slow waves and heartbeats. The methodology is illustrated on the single-subject level in Figure 4. After identifying individual slow waves, we studied the distribution of the eight closest heartbeats around each slow-wave onset. If there was no coupling, it would be expected that RS intervals should follow a uniform probability distribution, and that a time-averaged ECG would converge on a horizontal line around zero. As observed in previous work on cardio-respiratory coupling^28^, the distribution of time intervals between ECG R-waves and EEG slow wave (RS intervals; Figure 4B) was non-uniform and concentrated around specific phases in the slow-wave cycle. This appeared as a residual low-frequency oscillation in the ECG, after averaging around the slow-wave onset (Figure 4C); and as peaks in the distribution of heartbeat timings (Figure 4D).

**Figure 5:**
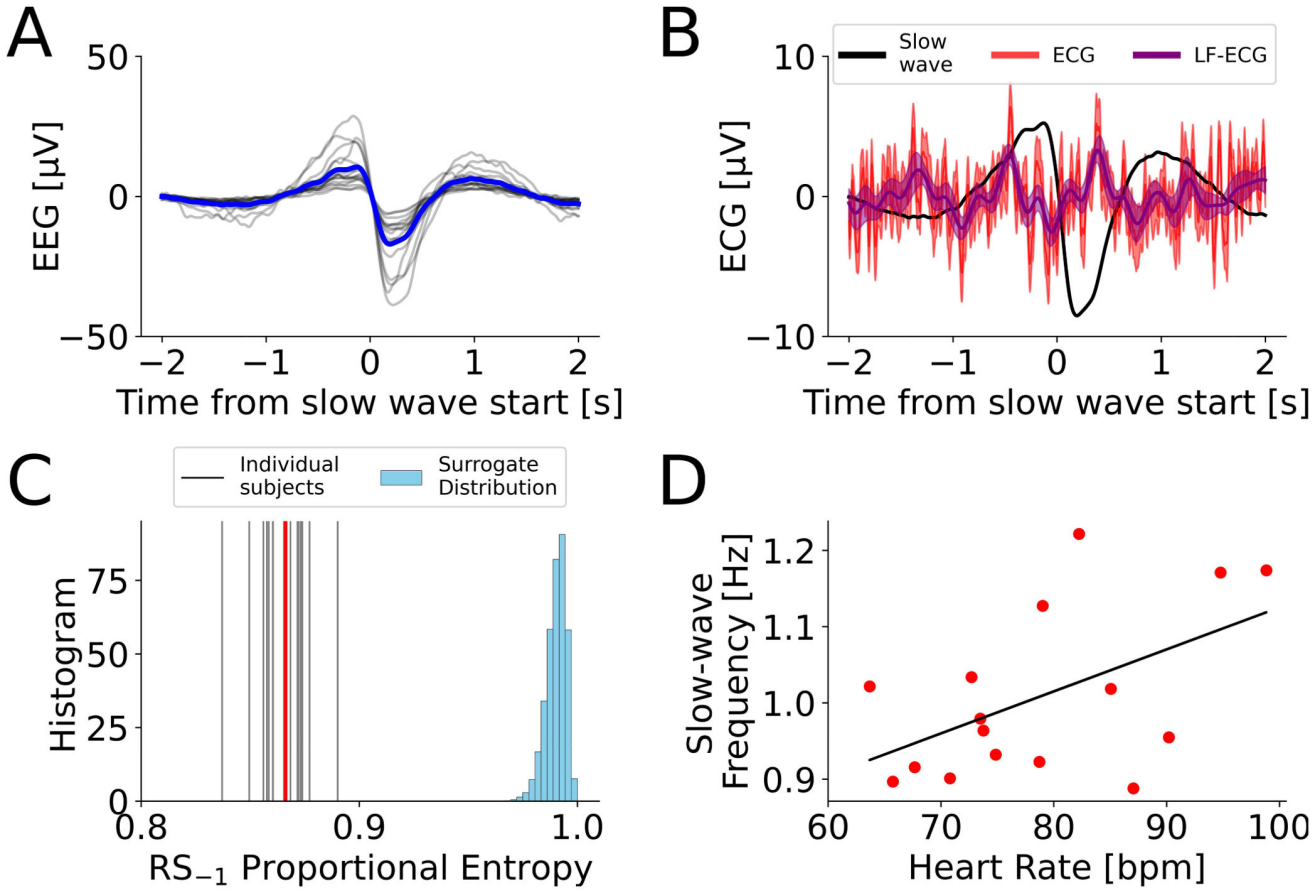
Group-level cortico-cardiac coupling in healthy volunteers. (A) Overall group-average slow wave (blue) with each subject’s mean in grey. (B) Group-average ECG (red = broadband, purple = 0.5Hz-4Hz only) time-locked to slow-wave (black) onset. The ongoing low-frequency ECG oscillation is also present at the group-level. (C) Slow-wave onset is significantly linked to the preceding heartbeat. The mean proportional Shannon entropy of intervals between the EEG slow-wave start and previous ECG R-wave across volunteers is shown in red, and the value for each of N=16 subjects is shown as a grey vertical line. The entropy of the surrogate distribution assuming random timings intervals between the EEG slow-wave start and previous ECG R-wave is in blue. (D) Individual heart rate and slow-wave frequency are related (Pearson r=0.519, P=0.0395).

Importantly, this effect was present and significant at the group level (Figure 5). The group-average lag between the ECG peak and EEG slow-wave onset was 447 [392, 510] ms (Figure 5B). The slow-wave/R-wave coupling, as measured by entropy in relation to a uniform null distribution was SH_P_=0.866±0.05 (P<0.001 compared to a uniform null hypothesis). Additional tests to verify this is not a random effect were carried out and can be found in the Supplemental Digital Content. Furthermore, at the group level, the subjects’ mean heart rates and slow-wave frequencies were significantly linearly correlated (Pearson r=0.519, P=0.0395).

The above analysis results were qualitatively unchanged when EEG data was re-referenced to linked mastoids (Supplementary Figure 3) and when ECG was time-locked to slow-wave trough instead of downward zero crossing (Supplementary Figure 4).

### Clinical dataset analysis

In order to explore whether the above heart rate results hold in a clinical setting, we analyzed the association between effect-site drug concentration and heart rate using a large general linear model with N=96 older patients collected as part of the AlphaMax trial (Table 2, Supplementary Figure 5). After adjusting for age, BMI, sex, and ASA status, all agents (propofol, fentanyl, and desflurane) had a significant effect on the heart rate (P<0.001). Propofol led to a mild increase in heart rate, on average with a coefficient of +1.3 bpm / (μg ml^-1^) (95%CI 1.1, 1.5). Fentanyl, however, led to a decrease in the heart rate, on average −2.6 (95%CI −2.7, −2.5) bpm / (ng ml^-1^), as did desflurane with average of −1.84 (95%CI −1.90, −1.78) bpm / (1%ET). The interaction terms were also significant though with smaller coefficients. With mean individual heart rate included as a regressor, no demographic parameters were significant, suggesting that the drug effects on the heart rate may be independent of these demographic variables. The effect size comparing HR with propofol <0.5μg/ml and >3μg/ml was Cohen’s d=0.796.

Interestingly, at the group level, mean intraoperative slow-wave frequency was not related to the mean heart rate in this dataset (Appendix 1, P=0.65). Furthermore, desflurane-fentanyl slow-wave frequency was significantly higher than propofol slow-wave frequency (propofol f=1.01±0.11Hz, desflurane-fentanyl f=1.26±0.15Hz (mean ± SD); Welch t-test P<0.0001), as seen before^31^. Unfortunately, individual ECG waveforms were not included in these data, so coupling between slow waves and individual heartbeats was not assessed.

## Discussion

### Propofol and the heart rate

In this paper, we have shown that administration of propofol leads to increased mean heart rate. The ultraslow propofol administration in healthy volunteers led to an increase in the mean heart rate of roughly +4 bpm/(μg/ml propofol concentration). In an exploratory analysis of an older patient population, the effect of propofol on heart was about three-fold smaller, on average +1.3 bpm/(μg/ml). These clear and significant mean heart rate increases confirmed our hypothesis but are surprising in view of the mixed existing literature.

Experimental studies have seen a heart rate increase across a variety of research paradigms^7,9–12,32^. We contend the lack of heart rate increase (or heart rate decrease) with propofol reported in some clinical studies may be due to other drugs given, the patient population, the surgical context, or dose-/rate-dependent effects. Clinically, it is common to administer opioids and other premedication, which can decrease the heart rate and affect cardiovascular dynamics^32,33^. The smaller heart rate increase observed in the older clinical population could be due to a previously proposed U-shape relationship between propofol and heart rate^11^. Older patients have higher anesthetic sensitivity and may be more susceptible to a heart rate decrease at relatively high propofol concentrations. This is supported by a previous healthy volunteer study where plasma concentrations of about 7.4μg/ml increased the heart rate by ~30bpm, but excessively high concentrations up to mean plasma levels of 18.3μg/ml reversed the effect and decreased the heart rate compared to lower concentrations^12^. Finally, clinical procedures may provide autonomic stimulation which could affect intrinsic heart rate increases with propofol.

The ultraslow induction rate used in our healthy volunteer study may also affect cardiac changes. This is supported by previous work finding rate-dependent cardiac effects of propofol with greater heart rate decrease in fast inductions, perhaps due to a rate-limiting central nervous system distribution process^19,34^. Our heart rate increase is unlikely to be due to anxiety, as whilst heart rate increased from baseline to loss of responsiveness, it continued to increase at the same rate when drug concentration increased beyond the point of loss of consciousness (Appendix 2). Finally, whilst it is plausible some of the heart rate increase could be due to endothelial irritation, no ‘pain on propofol injection’ phenomenon was reported by volunteers.

### Propofol and heart rate variability

The biological basis for the increase in mean heart rate may be due to propofol inhibiting cardioinhibitory vagal neurons in the brainstem^11^. Studies of propofol’s effect on autonomic cardiac influences have also produced mixed results.

The literature agrees that propofol reduces heart rate variability^6,12,35–37^, a result also confirmed in our experiment. The distinct sympathetic and parasympathetic contributions to this are less clear. An early study proposed that propofol mostly depresses sympathetic activity and suggested this as a mechanism for propofol bradycardia and hypotension. However, opioids were also used in that study^36^. Several studies since, including this one, have concluded that propofol predominantly decreases high-frequency heart rate variability. This is conventionally thought to reflect a decrease in parasympathetic vagal influences^6,12,35,37^. However, vagal and sympathetic activity tend to be mutually reciprocal, and thus we cannot conclusively show if the vagal decrease is a primary propofol effect, or be secondary to sympathetic activation. Notably however, the LF/HF ratio, traditionally taken as a measure of sympathetic/parasympathetic balance, had a much less consistent relationship with propofol concentration, indicating the sympathetic response was less consistent than the vagal response. We therefore conclude that propofol causes a shift in the autonomic balance to cause tachycardia, probably mainly via the parasympathetic branch, as this relationship was more consistent and more strongly correlated with propofol concentration. This is further supported by our R-wave/slow wave coupling results. These would not occur through slow sympathetic influences with a lag of up to tens of seconds.

This increased heart rate is unlikely to entirely be a reflex tachycardic response to vasodilation, as propofol has been shown to depress the baroreflex^38,39^. Specifically, reflex tachycardic responses to hypertension are reduced by propofol, both in conditions of normocapnia and hypercapnia^40^. In our study, as previously reported^8^, hypotension was not observed, and subjects had baseline end-tidal CO_2_ 39.3±3.3mmHg and peak end-tidal CO_2_ 47.1±5.7mmHg. A limitation of the present study is the lack of hemodynamic data, as tachycardia associated with decreased stroke volume due to propofol could be part of the heart rate increase mechanism. Future studies should focus on distinguishing between centrally and hemodynamically mediated mechanisms. A final potential confound is differing anesthetic susceptibility, and future studies could benefit from direct sampling of plasma propofol levels.

### Propofol and ECG morphology

We observed dose-dependent changes in ECG morphology that may reflect both direct cardiac effects and centrally-mediated changes of propofol. The R-wave amplitude decrease seen in our study might be related to previously observed propofol effects on ventricular depolarisation^42^. Propofol may decrease myocardial contractility, possibly due to a direct propofol effect on myocyte ability to expel intracellular calcium^43^, though this may only happen above clinical doses^44^. A change in the mean electrical axis or direct vagal effect could also explain the R-wave amplitude decrease; findings have been mixed so far^42^. Future studies should establish these changes in ventilated patients with more direct cardiac measurements such as cardiac output to assess the clinical significance of this finding.

### Cortico-cardiac coupling in propofol

Low-frequency cortico-cardiac coupling has been observed in sleep^16,45^. As propofol slow waves show some sleep-like properties^15^, we hypothesized this effect would also be present in anesthesia. We found individual cortical slow waves and cardiac R-waves were coupled as hypothesized. A heartbeat was most likely to precede the slow-wave onset by about 450ms, a time interval similar to that seen in sleep^16,45^. We did not see any evidence of dose-dependent coupling effects (Supplementary Figure 10).

Importantly, this coupling is non-trivial, as it relates to the phase relationship between individual EEG slow waves and the ECG, not just two ongoing oscillations that happen to have similar frequencies around 1Hz. The fact that the mean ECG line in figures 4D and 5C is not zero indicates a time-linked relationship – analogous to evoked potentials in EEG work.We explored this in detail through a simulation study, showing that our proportional entropy metric is sensitive to genuine coupling (Supplemental Digital Content Section 3).

Mensen et al. proposed several hypotheses for why this coupling may occur^16^. The first was a possible metabolic constraint. Overall, neurochemical tone favors hyperpolarized down states with heartbeats acting as a stimulus to evoke a down state when neuronal resources are depleted. Lower regional blood flow between heartbeats could have this effect on a few critical neurons leading to a network change. However, this seems unlikely as the necessary time resolution of changes in metabolic energy demand seems shorter than that of a damped feeder capillary blood flow, coupled with the energy substrate diffusion time and the presence of intrinsic neuronal energy stores. The other possibility is a third generator controlling both the heart rate and slow-wave genesis. Knowing this effect is present both in sleep and propofol anesthesia suggests a possible nature of this generator. Sleep and anesthesia differ in noradrenaline levels, but both show low acetylcholine levels^46^. Combined with the brainstem projecting both in a cephalad direction to higher brain areas and caudally to the heart, we propose it as a possible place for a common generator. For instance, the nucleus of the solitary tract or cholinergic pontine nuclei may project both to fast-spiking GABAergic interneurons in the thalamus and to medullar regions controlling the heart rate^47,48^. Given that the thalamus is involved in slow-wave generation *in vivo*, a brainstem connection could explain cortico-cardiac coupling, perhaps by weak-coupling synchronization^49,50^. This is supported by subjects with a faster heart rate also having faster slow-wave frequency. Interestingly, this frequency relationship was not observed during desflurane-fentanyl slow waves (Appendix 1), suggesting volatiles may differ in their cortico-cardiac coupling effects. Further work is needed to explain the relationship between slow waves and cardiac activity, especially as pertains to wider coupling of autonomic and central activity^17,18^. Our proposed common brainstem generator could be ruled out if patients with pacemakers also show this coupling.

In summary, in this observational study, slow propofol administration in healthy subjects robustly led to an increase in mean heart rate that was strongly proportional to drug concentration, and not influenced by changes in behavioral responsiveness. A preliminary analysis in a larger clinical dataset replicated this result, but with a decreased effect size. The heart rate increase could be explained with decreased parasympathetic inputs, as indexed by decreased high-frequency heart rate variability. Similarly to sleep, frontal cortical slow waves preferentially occurred coupled to the heart rhythm, perhaps due to a common brainstem generator. The observational nature of the study limits causal inferences that can be made, and more work is needed to elucidate the mechanism and role of these cardiac changes and the clinical significance of their coupling to the cortex.

Thus, in the clinical management of patient hemodynamics, propofol should not be assumed to decrease the heart rate. In fact, particularly for slow infusions and younger patients, propofol is likely to increase the heart rate. Ultimately, heart rate will be a complex result of opioid, hypnotic, and surgical factors.

## Supplementary Material

Appendix 1

Appendix 2

Supplementary Material

## Figures and Tables

**Table 1:** Summary of datasets used in this study.

Dataset name	N	Age / years median (range)	Sex	ASA Status	Concomitant mediations
**Volunteer propofol^8^**	16	28.5 (19-43)	8F/8M	I	No
**Patients AlphaMax^20^**	96	74 (61-86)	30F/66M	II, III	Fentanyl, desflurane

**Table 2:** General linear model coefficients for heart rate in the AlphaMax clinical study. Lower / Upper columns indicate 95% confidence intervals. The model confirms propofol’s tendency to increase the heart rate independent of other regressors, unlike fentanyl and desflurane.

Name	Estimate	Lower	Upper	Standard Error	t-statistic	P-Value
**Intercept**	45.444	17.620	73.267	14.196	3.201	**0.001**
**Age**	0.213	-0.142	0.568	0.181	1.177	0.239
**BMI**	0.122	-0.288	0.532	0.209	0.584	0.559
**ASA**	1.976	-2.853	6.806	2.464	0.802	0.423
**Sex = F**	0.367	-4.181	4.915	2.320	0.158	0.874
**Propofol [μg/ml]**	1.319	1.120	1.517	0.101	13.016	**<0.001**
**Fentanyl [ng/ml]**	-2.604	-2.706	-2.501	0.052	-49.682	**<0.001**
**Desflurane [%]**	-1.838	-1.895	-1.780	0.029	-62.566	**<0.001**
Propofol*Fentanyl	0.988	0.946	1.030	0.021	46.510	**<0.001**
Propofol*Desflurane	-0.711	-0.766	-0.656	0.028	-25.266	**<0.001**
Fentanyl*Desflurane	0.463	0.437	0.489	0.013	35.018	**<0.001**

## Data Availability

The datasets analyzed during the current study are available from the corresponding author on reasonable request via https://zenodo.org/record/1168447.
