# Supplementary material for: Effect of propofol on heart rate and its coupling to cortical slow waves in humans
Source: Anesthesiology. Author manuscript; Available in PMC 2024 Jan 1. (PMC7615371; doi:10.1097/ALN.0000000000004795)
Supplement: Appendix 1 [file EMS189056-supplement-Appendix_1.docx]

**APPENDIX 1**

**Slow-wave Frequency in Clinical Data**

**
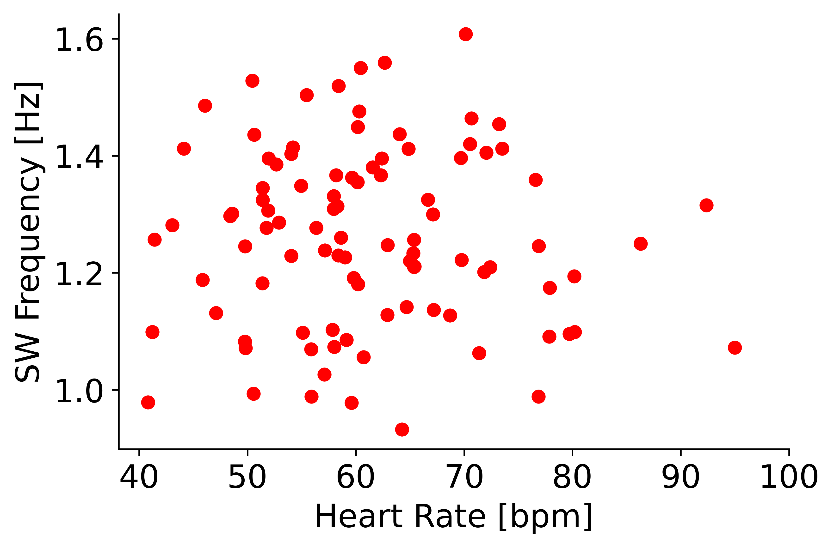
**

**Supplementary Figure 1:** Mean slow-wave frequency and heart rate are not related during clinical desflurane-fentanyl intraoperative general anesthesia (N=96 patients, Pearson r=-0.05, P=0.65).
