# Supplementary material for: Effect of propofol on heart rate and its coupling to cortical slow waves in humans
Source: Anesthesiology. Author manuscript; Available in PMC 2024 Jan 1. (PMC7615371; doi:10.1097/ALN.0000000000004795)
Supplement: Appendix 2 [file EMS189056-supplement-Appendix_2_.docx]

**APPENDIX 2**

**Heart Rate around Loss of Responsiveness in Volunteer Data**

**
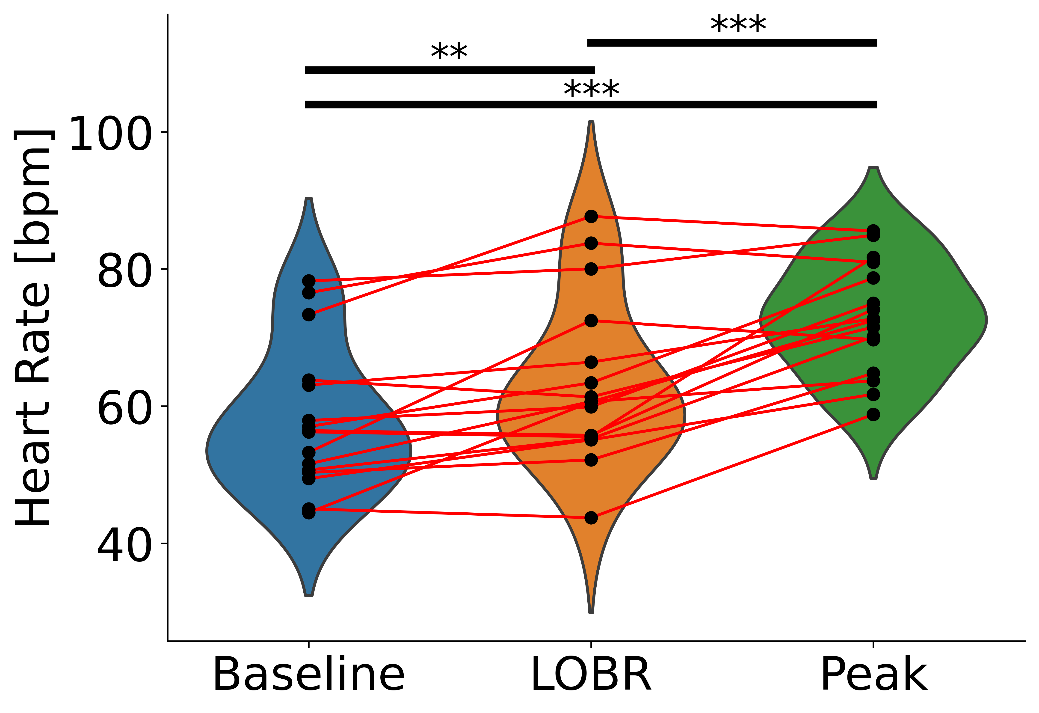
**

**Supplementary Figure 2:** Heart rate in ultra-slow propofol infusion increases from baseline to loss of behavioral response to auditory and laser pain stimuli (LOBR) and peak anesthesia. ** indicates Bonferroni-corrected paired t-test P<0.01, *** indicates P<0.001.
