# Supplementary material for: Effect of propofol on heart rate and its coupling to cortical slow waves in humans
Source: Anesthesiology. Author manuscript; Available in PMC 2024 Jan 1. (PMC7615371; doi:10.1097/ALN.0000000000004795)
Supplement: Supplementary Material [file EMS189056-supplement-Supplementary_Material.pdf]

SUPPLEMENTAL DIGITAL CONTENT

1. Robustness analyses

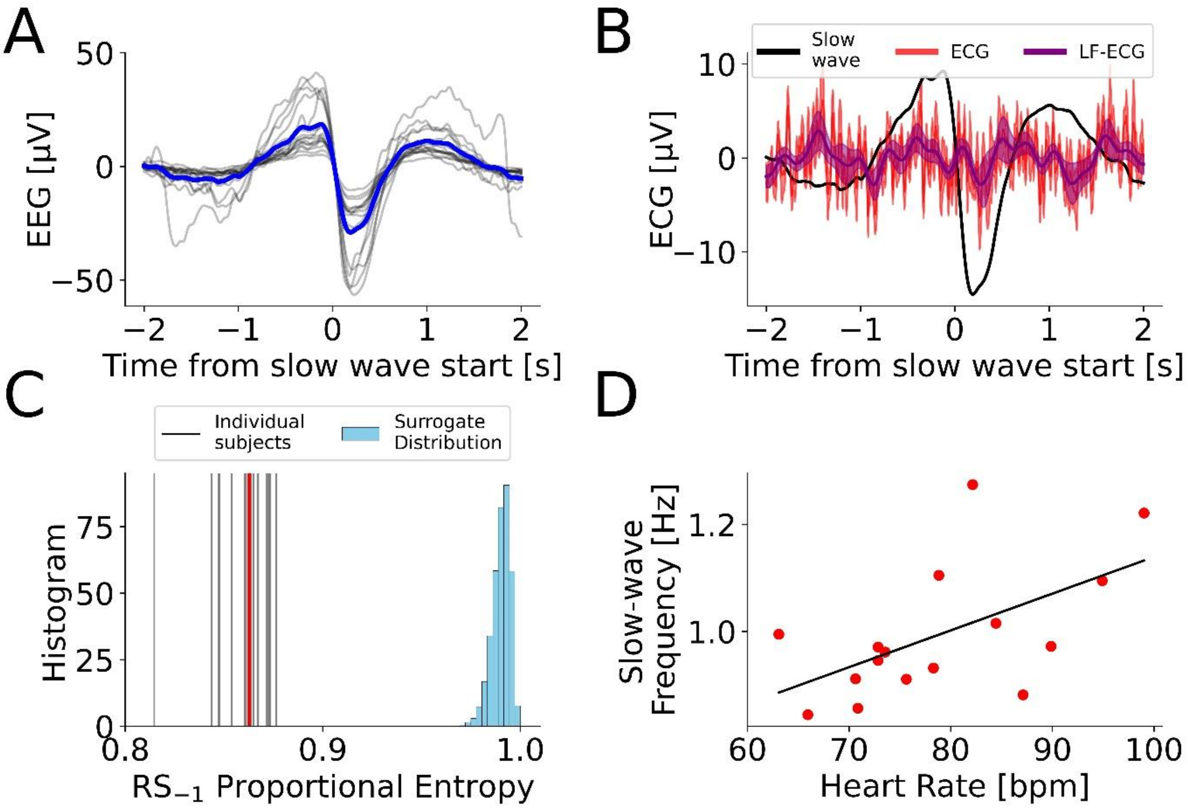

Supplementary Figure 3: Cortico-cardiac coupling results are similar using linked mastoid referencing. Panels are the same as in Figure 5 in the Main Text.

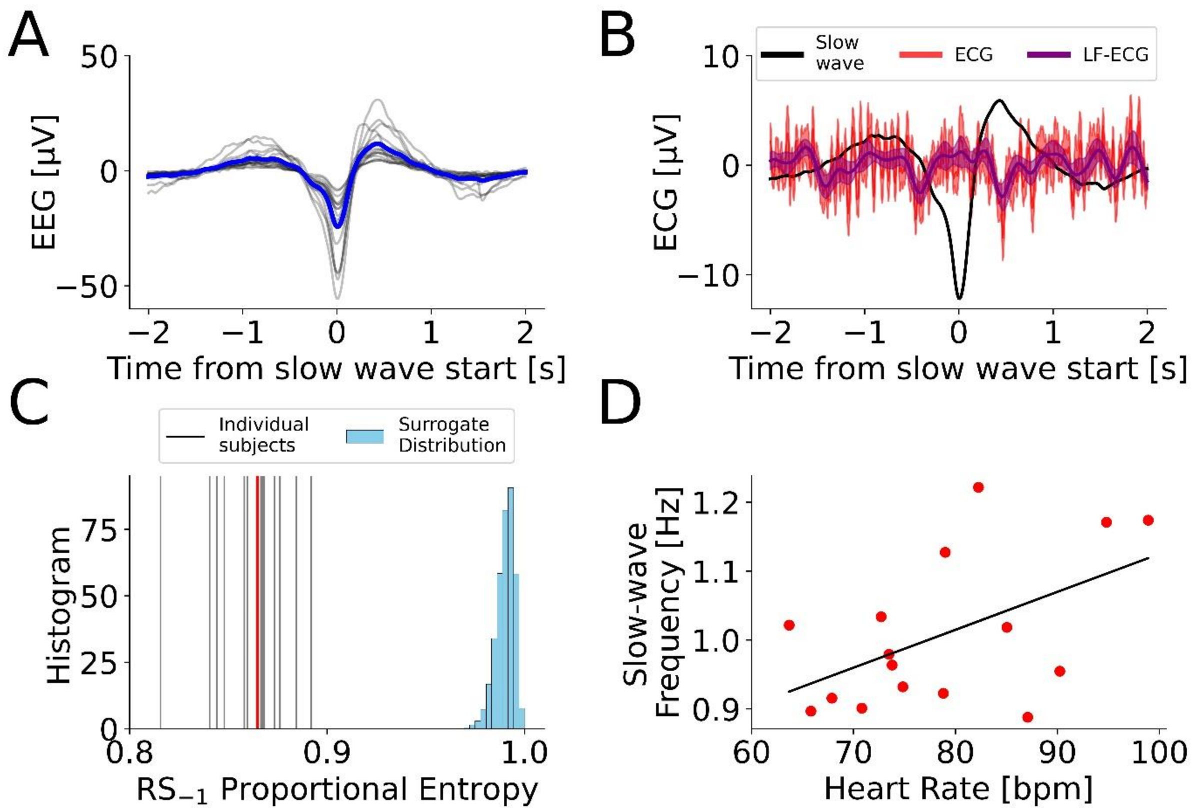

Supplementary Figure 4: Cortico-cardiac coupling results are similar when time-locking to EEG slow-wave troughs rather than downward zero crossings. Panels are the same as in Figure 5 in the Main Text.

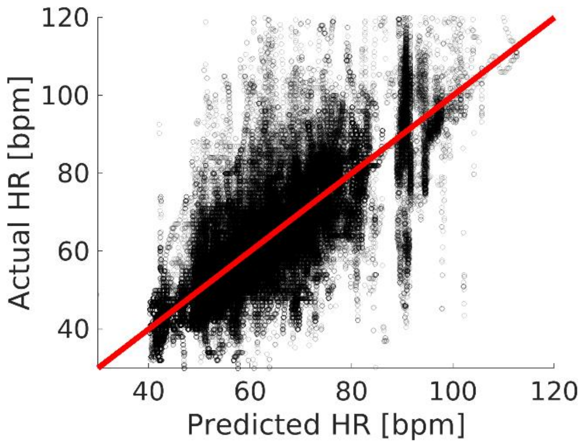

Supplementary Figure 5: Observed heart rate (HR) vs that predicted by a general linear model in an older, clinical population (see Main Text for model details).

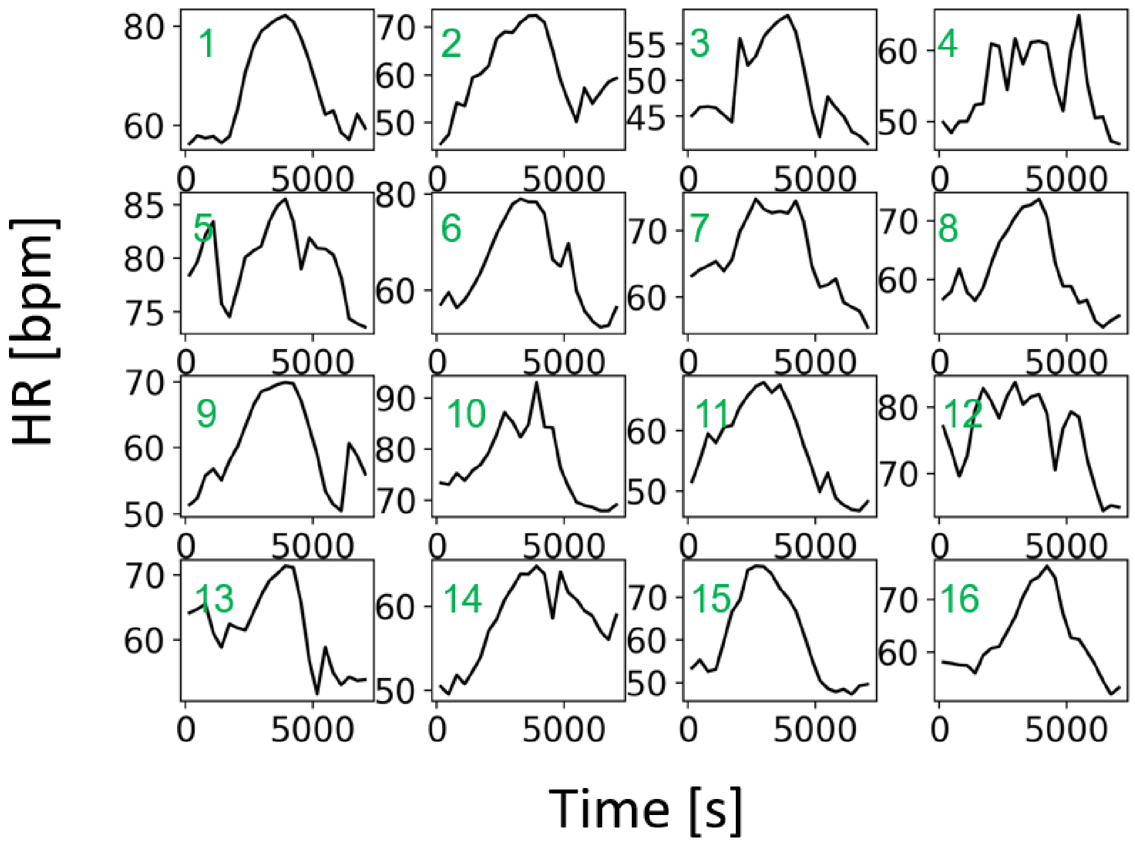

Supplementary Figure 6: Observed heart rate (HR) in each individual subject from the volunteer study (subject number in top-left). The HR increase seen in the main text was demonstrated in every subject.

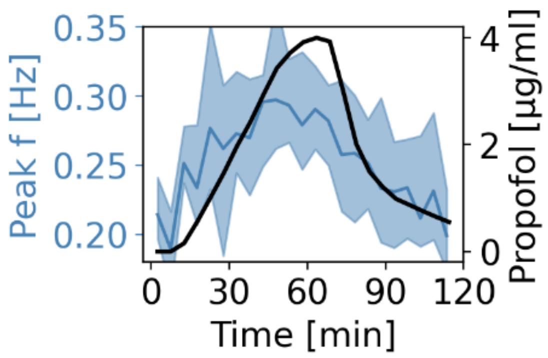

Supplementary Figure 7: Peak frequency in the high-frequency heart rate variability band that indexes the respiratory vagal peak (blue; median ± 95% CI) tracks propofol concentration (black).

2. Further tests of cortico-cardiac coupling

In the Main Text, the method introduced by Galletly et al[49] was used to test the presence of coupling against a uniformly random distribution. Here, we provide alternative tests to verify the robustness of this result.

First, the Galletly et al method only tests RS_{-1} intervals. We wanted to test for the presence of an ongoing oscillation, not just RS_{-1}. To test this in each subject and at the group-mean level, a histogram of RS timings (range -3s to 3s) was computed and adjusted for between-subject heart rate differences by multiplying it by the mean heart rate across the entire experiment for each subject. Next, the histogram's autocorrelation calculated. Significant, sine-like autocorrelation would signify an ongoing ECG oscillation around the slow-wave onset. To assess significance, autocorrelation for the same histogram but with uniformly random RS timings in the (-3s, 3s) interval was computed for N=1000 surrogate distributions. To further assess whether autocorrelation showed sinusoidal behavior, and to find the delay whether this possible ECG oscillation and the slow wave, an exponentially decaying sinusoid was fitted to it (scipy.optimize.curve_fit) and significance established using the Bartlett test on residuals compared to the mean. This revealed the presence of an ongoing oscillation at the group level (Supplementary Figure 8), present strongly in 10 out of 16 subjects (Supplementary Figure 9). In these subjects, the mean P-value for a sinusoidal fit was P=0.0012 with R^2=0.723±0.056. On the group level across all participants, the distribution of heartbeat timings in relation to the slow-wave onset showed significant autocorrelation with P<0.001 and R^2=0.857 to a decaying sinusoid fit with exponential half-life of 2.51s (Supplementary Figures 8C, D).

Next, we wanted to know if the strength of coupling varies with dose or is a general slow-wave phenomenon (Supplementary Figure 10). We plotted the proportional entropy SH_P across time. No significant effect was observed (P>0.5).

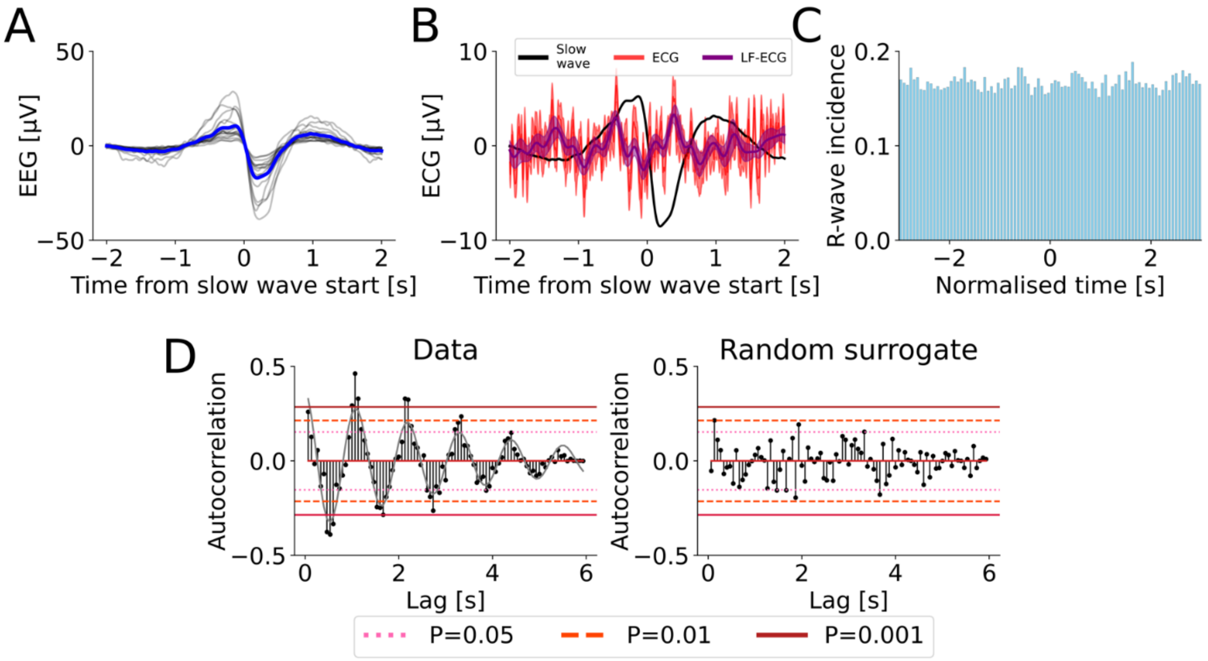

Supplementary Figure 8: Group-level cortico-cardiac coupling in healthy volunteers. (A) Mean slow wave detected (blue) with each subject mean in grey. (B) Group-average ECG (red – broadband, purple – 0.5Hz-1.5Hz only) time-locked to slow-wave (black) onset. The ongoing low-frequency ECG oscillation is present at the group-level. (C) Group-mean histogram of R-wave timings relative to slow-wave onset, with time for each subject adjusted for their mean heart rate. Individual heartbeats preferentially occur in phase with the slow wave. (D) Autocorrelation of (C) shows clear oscillatory structure (left). This is significant compared to P-values derived from N=1000 uniformly random surrogate timing distributions.

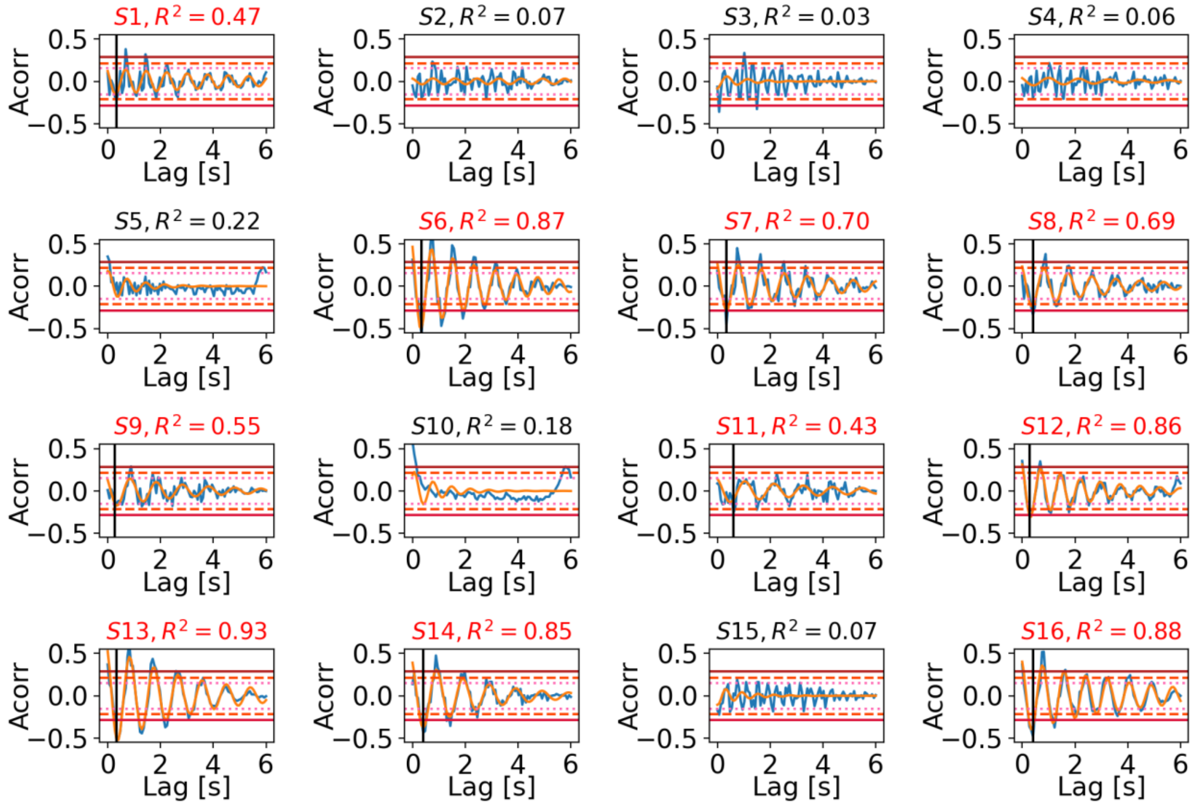

Supplementary Figure 9: Autocorrelation of RS histograms in each participant (blue) and a decaying sine fit (orange). 10 out of 16 participants showed a significant ongoing low-frequency ECG oscillation (red titles).

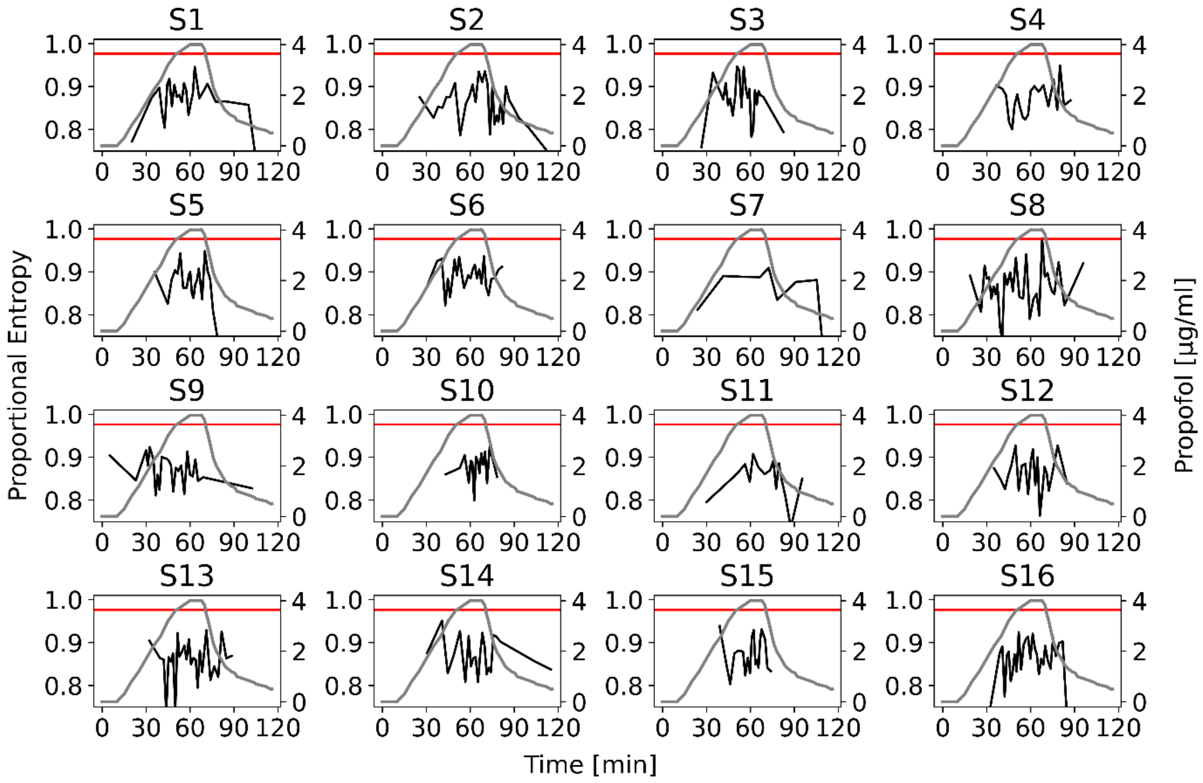

Supplementary Figure 10: Dose-dependence of cortico-cardiac coupling. Lower entropy means more significant coupling between slow-wave onset and the preceding heartbeat. No changes were observed between different propofol doses.

3. Understanding cortico-cardiac coupling

We performed extensive simulations to understand where the coupling may come from. Naively, one may wonder if two oscillations both around 1Hz (heartbeat and slow waves) trivially appear coupled because of their similar frequency. To study this, we simulated 10 minute EEG and ECG recordings with 100Hz sampling rate. The ECG was simulated with the neurokit2 module to have HR=60bpm (SD 1bpm). The EEG was simulated as the sum of aperiodic noise with spectral exponent -1.5 (amplitude 10µV), a bursty alpha oscillation (frequency 10Hz, probabilities to enter and exit burst 0.5, amplitude 5µV), and different types of slow waves.

First, slow waves were modelled as a bursty oscillation at 1Hz, perfectly coherent with the heart rate. Trivially, this causes high coupling (Supplementary Figure 11A, entropy=0.502). Next, slow waves were kept at 1Hz, but were insterted at random times. This destroys the coupling despite having the same frequency (Supplementary Figure 11B, entropy=0.982). Then, slow waves were again made a coherent oscillation, but this time at 1.1Hz. This also makes the coupling much weaker (Supplementary Figure 11C, entropy=0.981). Finally, the 1.1Hz slow waves were inserted every 3s. This recovers the coupling despite different frequencies of slow waves and heart rate (Supplementary Figure 11D, entropy=0.650).

In short, unless the frequency of heart rate and slow waves is exactly in an integer relationship, low entropy points to each slow wave being significantly coupled to a heartbeat. This is not a trivial effect and demands an explanation as per the Main Text.

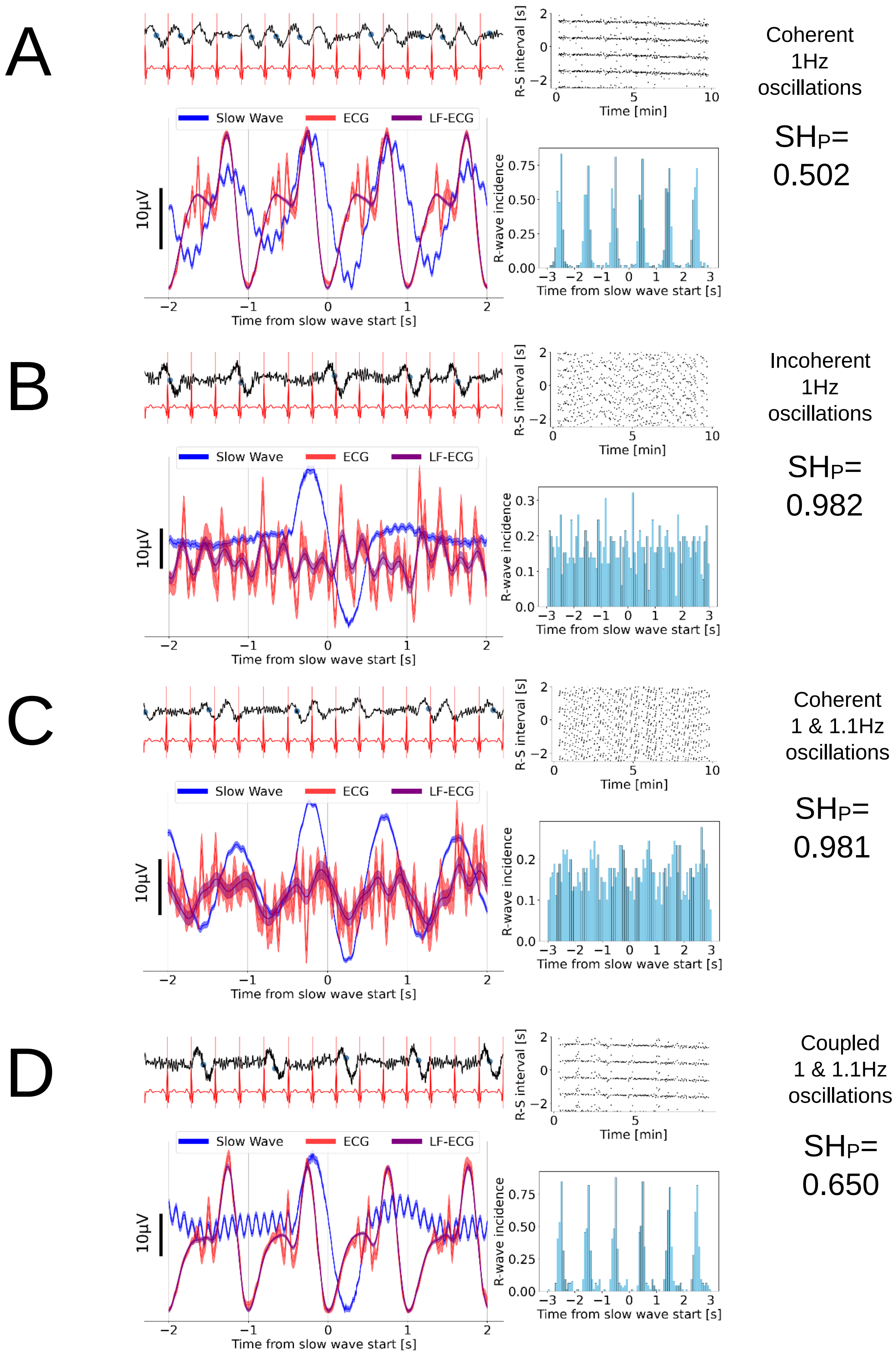

A
Coherent
1Hz
oscillations
SHP=
0.502
B
Incoherent
1Hz
oscillations
SHP=
0.982
C
Coherent
1 & 1.1Hz
oscillations
SHP=
0.981
D
Coupled
1 & 1.1Hz
oscillations
SHP=
0.650
Slow Wave
ECG
LF-ECG
10µV
Time from slow wave start [s]
R-S interval [s]
Time [min]
R-wave incidence

Supplementary Figure 11: Explaining coupling between slow waves and R-waves. (A) Two ongoing 1Hz oscillations with constant phase difference are trivially coupled. (B) Coupling is destroyed if slow waves occur at random times, even if both oscillations are at 1Hz. (C) Coupling is also much weaker if frequencies do not match even slightly, here 1Hz for heartbeats and 1.1Hz for ongoing slow waves. (D) Coupling is restored if each slow wave is related to individual heartbeats, even if their frequency is different.
